# Spectrum of Dystonia in Progressive Supranuclear Palsy: A Prospective Observational Study and Review of Literature

**DOI:** 10.5334/tohm.1243

**Published:** 2026-08-19

**Authors:** Kartika Gulati, Sanjay Pandey

**Affiliations:** 1 Department of Neurology and Stroke Medicine, Amrita Institute of Medical Sciences, Mata Amritanandamayi Marg Sector 88, Faridabad, Delhi National Capital Region, India

**Keywords:** Progressive supranuclear palsy, dystonia, corticobasal syndrome

## Abstract

**Background:**

Dystonia is an under-recognized yet clinically significant feature of Progressive supranuclear palsy (PSP), contributing to disability and impaired motor function.

**Method:**

In this prospective study, we examined the clinical and neuroimaging details of eighty-eight consecutive PSP patients who visited our movement disorder clinic [2024–2025]. Patients fulfilling the 2017 Movement Disorder Society-PSP criteria for possible or probable PSP were enrolled after ethics approval and informed consent. We aimed to evaluate the prevalence, phenomenology, subtype associations, clinical and neuroimaging correlates of dystonia. Additionally, a literature search was performed and 29 articles were included for detailed review.

**Result:**

The literature review identified predominantly retrospective studies, reporting dystonia in approximately 30–60% of the patients, with variable patterns of cranio-cervical and limb involvement. In our prospective cohort, dystonia was present in 64.8% (n = 57) cases. PSP-RS was the most frequent subtype (39.8%), followed by PSP-P (26.1%) and PSP-CBS (23.8%). Dystonia was more common in PSP-RS and PSP-CBS, with predominant limb dystonia in PSP-CBS, and cranio-cervical dystonia in PSP-RS. A trend toward earlier dystonia was observed in PSP-CBS. Patients with dystonia demonstrated lower Midbrain to pons ratios and higher Magnetic resonance parkinsonism index, suggesting greater brainstem involvement. Botulinum toxin therapy resulted in clinical improvement in selected dystonia patients.

**Conclusion:**

Dystonia is a frequent and clinically meaningful feature in PSP. Our prospective study expands upon the existing literature by demonstrating distinct subtype-specific patterns and neuroimaging correlates, supporting the concept of a “dystonia spectrum” in PSP and highlighting its relevance for clinical recognition and management.

## Introduction

Progressive Supranuclear Palsy (PSP) is a sporadic neurodegenerative disorder within the spectrum of parkinsonism, pathologically defined by the accumulation of 4-repeat tau [1]. Classically, it presents with an akinetic-rigid syndrome, early falls, supranuclear gaze palsy, and pseudobulbar features. The global prevalence is estimated at around 5–6.4 per 100,000 population, although this varies depending on the diagnostic criteria and healthcare settings [2]. The mean age at onset is approximately 63 years, with a median survival of nearly 7 years from symptom onset [3].

Although the clinical spectrum of PSP has broadened considerably, it is still often described in terms of its classical presentation, overlooking the diversity of its subtypes [4,5]. The 2017 Movement Disorders Society (MDS) criteria classify PSP into distinct variants based on four functional domains: ocular motor dysfunction, postural instability, akinesia, and cognitive dysfunction [6]. These are further stratified into levels of diagnostic certainty, supported by clinical features and imaging markers [7]. While definitive diagnosis requires neuropathological confirmation, the use of these criteria has improved the recognition of non-classical PSP variants with greater sensitivity and specificity [8,9,10].

PSP involves multiple brain regions, particularly the subthalamic nucleus, globus pallidus, substantia nigra, and brainstem structures, with the distribution of pathology influencing the clinical phenotype. The histopathological hallmark is the presence of tau-positive globose neurofibrillary tangles, reflecting widespread 4R-tau pathology. Involvement of multiple neurotransmitter systems and both pre- and post-synaptic pathways contributes to the limited and often short-lived response to pharmacological therapies [11].

Neuroimaging markers such as the midbrain-to-pons ratio and the magnetic resonance parkinsonian index (MRPI) are useful in supporting the diagnosis of PSP and differentiating it from other parkinsonian disorders [12,13].

Dystonia is a recognized feature of PSP, affecting both limb and axial musculature [14,15]. Previous studies have described its prevalence and clinical characteristics in atypical parkinsonian syndromes [16,17,18,19]. Despite this, dystonia remains under-emphasized in routine clinical descriptions, often overshadowed by more prominent features such as falls and gaze palsy. Moreover, much of the existing literature is based on retrospective analyses and small cohorts, with limited focus on variation across PSP subtypes or on associated clinical and imaging correlates. A systematic characterization of dystonia is clinically relevant because its phenomenology may provide valuable clues for recognizing PSP subtypes, particularly in patients with non-classical presentations. Furthermore, identifying subtype-specific patterns and clinical correlates of dystonia may facilitate earlier and more accurate diagnosis. This, in turn, may enable timely implementation of targeted symptomatic therapies, such as botulinum toxin treatment, ultimately improving patient care and functional outcomes.Thus, the present study was undertaken to systematically evaluate dystonia in PSP, including its prevalence, phenomenology, anatomical distribution, subtype associations, clinical and imaging correlates, and therapeutic implications.

## Methods

This prospective study was conducted at our tertiary care teaching institute, Amrita Institute of Medical Sciences, Faridabad, India, [2024–2025]. The study was approved by the Institutional Ethics Committee. Patients attending our Movement disorder clinic were clinically examined and those diagnosed as possible or probable PSP based on the 2017 International Parkinson and MDS-PSP study group diagnostic criteria were included in the study [6]. Written informed consent was obtained from all patients for the publication of their videos. PSP patients were subclassified into variants such as PSP-RS (Richardson’s syndrome), PSP-P (parkinsonism), PSP-F (frontal), PSP-PGF (progressive gait freezing), PSP-CBS (cortico-basal syndrome), PSP-PLS (primary lateral sclerosis), PSP-C (cerebellar ataxia), PSP-SL (speech/language disorders), PSP-PI (postural instability), and PSP-OM (oculomotor dysfunction). The cases were classified based on the clinical assessment done by two movement disorder specialists [KG, SP]. Patients fulfilling the 2017 MDS diagnostic criteria for ‘probable’, ‘possible’, or ‘suggestive of’ PSP were included. Clinical phenotypes were assigned according to the corresponding MDS criteria. Demographic data such as age of onset, gender, disease duration, clinical characteristics, and PSP subtype were collected. Presence of dystonia, anatomical distribution, timing of dystonia, and characteristics across various PSP subtypes were recorded. MRI findings such as midbrain to pons ratio, magnetic resonance parkinsonism index (MRPI) and cortical atrophy patterns were recorded. The data was entered into an MS Excel sheet and statistically analysed.

**Figure 1:**
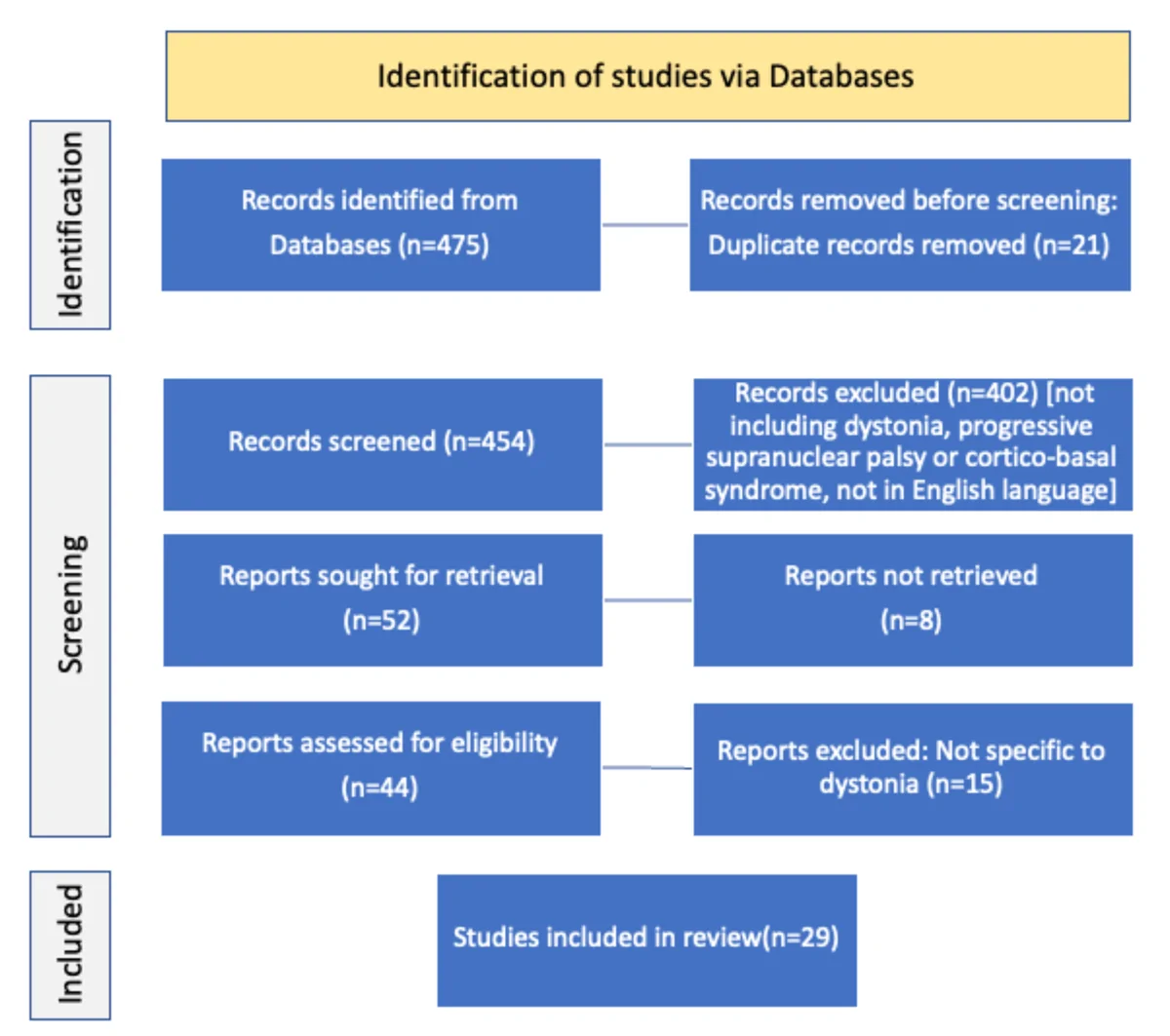
Search strategy for dystonia in Progressive supranuclear palsy.

In addition, a literature review was performed to evaluate the spectrum of dystonia in PSP. We searched PubMed on 8^th^ May 2026 and identified 271 articles on ‘Dystonia AND Progressive supranuclear palsy’, and 204 articles on ‘Dystonia AND Corticobasal syndrome’. The search query combined these terms using: articles containing “Dystonia” [Mesh] OR “Dystonia” [Title/Abstract]) AND (“Progressive supranuclear palsy” [Mesh] OR “Progressive supranuclear palsy” [Title/Abstract] OR “Corticobasal syndrome” [Mesh] OR “Corticobasal syndrome” [Title/Abstract] were included. After removal of duplicates, all relevant English-language articles were screened. Studies lacking details regarding dystonia, not focused on PSP or CBS, or published in languages other than English were excluded. A total of 29 articles were included in the detailed review (see Figure 1).

## Statistical analysis

Statistical analyses were performed using IBM SPSS Statistics (IBM Corp., Armonk, NY). Continuous variables were expressed as mean ± standard deviation (SD) or median (interquartile range [IQR]), depending on the distribution, assessed using the Shapiro-Wilk test. Comparisons between PSP patients with and without dystonia were conducted using Welch’s independent samples t-test to account for unequal variances and group sizes. Continuous variables across major PSP subtypes (PSP-RS, PSP-P, and PSP-CBS) were compared using one-way analysis of variance (ANOVA) for normally distributed data, and the Kruskal–Wallis test for non-normally distributed variables. Subtypes with very small sample sizes (n ≤ 4) were excluded from inferential statistical comparisons and are described descriptively. Categorical variables, including gender, subtype distribution, and presence of early dystonia (onset of dystonia <= 12 months), were analysed using the Chi-square test of independence or Fisher’s exact test where appropriate. All tests were two-tailed, and a p-value < 0.05 was considered statistically significant.

## Results

### Demographics

88 patients were included in the study. The study population comprised of 34.1% females (n = 30) and 65.9% males (n = 58). The mean age at onset was 65.9±7.4 years (range 50–85), and the mean symptom duration was 3.8 ± 2.3 years (range 0.5–12).

### Clinical features

Dystonia was a prominent clinical feature in our cohort, affecting 64.8% of the patients (n = 57). The most frequent clinical features at presentation were an akinetic-rigid syndrome (86.4%, n = 76), along with oculomotor dysfunction (68.2%, n = 60) and postural instability (58%, n = 51). These features were not mutually exclusive.

Tremor was present in 37.5% of the cases (n = 33), most commonly as an action tremor (n = 24), followed by rest tremor (n = 5) and four patients exhibiting both action and rest tremor.

A substantial proportion of patients (31.8%, n = 28) exhibited features consistent with sitting apraxia, characterized by impaired motor planning during the act of sitting. Clinically, this manifested as misjudging chair position or depth, perching at the edge of the seat, or failing to appropriately align the body before sitting despite adequate limb strength. Features suggestive of REM sleep behaviour disorder were observed in 12.5% (n = 11).

### PSP Subtype Distribution

PSP-RS was the most frequent subtype (39.8%, n = 35), followed by PSP-P (26.1%, n = 23) and PSP-CBS (23.8%, n = 21) (see Table 1). Less frequent subtypes included PSP-F (n = 4), PSP-PGF (n = 2), PSP-C (n = 2) and PSP-SL (n = 1). According to the 2017 MDS diagnostic criteria, 46 patients fulfilled ‘probable’ PSP, 31 fulfilled ‘possible’ PSP, and 11 fulfilled ‘suggestive of’ PSP.

**Table 1:** Distribution of Progressive supranuclear palsy subtypes in the study cohort.


SUBTYPE (NUMBER OF CASES)	MEAN ±SD AGE OF ONSET(YEARS)	GENDER (M/F)	MEAN ± SD DISEASE DURATION (YEARS)	MEAN ±SD MDS-UPDRS PART III SCORE	MEAN ± SD MOCA SCORE	DYSTONIA %	MEAN ± SD TIME TO DYSTONIA (MONTHS)	TREMOR %	MEAN ± SD M:P	MEAN ± SD MRPI

PSP-RS (35)	64.8 ± 7.7	63% M, 37% F	3.9 ± 2.4	33.2 ± 4.8	16.1 ± 4.2	82.9% (n = 29)	28.8 ± 21.2	22.9% (n = 8; action tremor)	0.17 ± 0.1	19.1 ± 6.7

PSP-P (23)	67.1 ± 7.8	78% M, 22% F	4 ± 2.6	30.1 ± 5.6	22.1 ± 2.2	39.1% (n = 9)	30.0 ± 18.7	69.6% (n = 16; rest tremor 4, action tremor 8, both rest and action tremor 4)	0.28 ± 0.0	10.3 ± 4.0

PSP-CBS (21)	65.7 ± 5.7	62% M, 38% F	4 ± 1.9	27.5 ± 5.4	17.3 ± 4.4	85.7% (n = 18)	19.1 ± 19.2	23.8% (n = 5; rest tremor 1, action tremor 4)	0.26 ± 0.1	12.2 ± 6.0

PSP-F (4)	72.3 ± 10.5	100% M	1.8 ± 0.5	29 ± 5.3	14.5 ± 4	NA	NA	50% (n = 2; action tremor)	0.28 ± 0.2	14.7 ± 0.0

PSP-PGF (2)	63.3 ± 2.5	100% F	2.3 ± 2.5	28 ± 8.5	24 ± 1.4	50% (n = 1)	NA	50% (n = 1; action tremor)	0.36 ± 0.1	12.3 ± 1.3

PSP-C (2)	63.5 ± 2.1	50% each	4 ± 2.8	29 ± 1	22 ± 2.8	NA	NA	50% (n = 1; action tremor)	0.33 ± 0.1	NA

PSP-SL (1)	72 ± 0	100% F	5 ± 0	18 ± 0	19 ± 0	NA	NA	0%	0.4 ± 0.0	NA


**Footnotes:** PSP Progressive supranuclear palsy, PSP-RS Richardson’s syndrome, PSP-P Parkinsonism, PSP-F Frontal, PSP-PGF Progressive gait freezing, PSP-CBS Cortico-basal syndrome, PSP-C Cerebellar ataxia, PSP-SL Speech/language disorders, SD Standard deviation, M Male, F Female, MDS-UPDRS Movement Disorder Society-Unified Parkinson’s Disease Rating Scale, MoCA Montreal Cognitive Assessment, M:P Midbrain to pons ratio, MRPI Magnetic Resonance Parkinsonism Index, NA Not applicable.

There were no significant differences among PSP-RS, PSP-P, and PSP-CBS with respect to age at onset (p = 0.41), disease duration (p = 0.98), or gender distribution (p = 0.36).

Motor severity, assessed using the MDS-UPDRS Part III, differed significantly across subtypes (p = 0.002), with PSP-RS demonstrating higher motor scores compared to PSP-P and PSP-CBS. Cognitive performance also varied significantly. Patients with PSP-P had higher Montreal Cognitive Assessment (MoCA) and Frontal Assessment Battery (FAB) scores compared to PSP-RS and PSP-CBS (both p < 0.001), indicating relatively preserved cognition in this subgroup.

Tremor was most frequently observed in PSP-P (n = 16), followed by PSP-RS (n = 8) and PSP-CBS (n = 5), with occasional cases in other subtypes. Sitting apraxia was most frequently observed in PSP-RS (n = 19), followed by PSP-CBS (n = 6) and PSP-P (n = 3). Features suggestive of REM sleep behaviour disorder were more commonly seen in PSP-P (n = 6), followed by PSP-CBS (n = 3) and PSP-RS (n = 2).

Cortical features were prominent in PSP-CBS, with limb apraxia present in 76.1% (n = 16) and alien limb phenomenon in 47.6% (n = 10) (see Videos 1 and 2). Of these, three cases were the anterior subtype (groping behaviour), while seven were the posterior subtype, characterized by limb foreignness or levitation. Myoclonus was observed in 28.6% (n = 6) of PSP-CBS cases and was stimulus-sensitive.

**Video 1:** A patient with Progressive Supranuclear Palsy–Corticobasal Syndrome (PSP-CBS), demonstrating groping behaviour and left upper limb dystonia consistent with alien limb phenomenon.

**Video 2:** A patient with Progressive Supranuclear Palsy–Corticobasal Syndrome (PSP-CBS) demonstrating left upper limb dystonia, myoclonus, and alien limb phenomenon.

### Neuroimaging

Significant differences in neuroimaging markers were observed across PSP subtypes. The midbrain-to-pons (M:P) ratio differed significantly among PSP-RS (0.17 ± 0.10), PSP-P (0.28 ± 0.00), and PSP-CBS (0.26 ± 0.10) (p < 0.001), with lower values in PSP-RS indicating greater midbrain atrophy. Similarly, MRPI values varied significantly (p < 0.001), with PSP-RS demonstrating the highest values (19.1 ± 6.7), compared to PSP-P (10.3 ± 4.0) and PSP-CBS (12.2 ± 6.0), consistent with more pronounced brainstem involvement.

Among PSP-CBS cases, 11 of 21 demonstrated parietal cortical atrophy contralateral to the side of dystonia. Cerebellar atrophy was observed in one patient with the PSP-C subtype. Subtypes with small sample sizes (n ≤ 4) were excluded from inferential statistical analysis.

### Dystonia

Dystonia was present in 64.8% of patients (n = 57). Age at onset, gender distribution, disease duration, motor severity (MDS-UPDRS Part III), and cognitive scores (MoCA) were comparable between patients with and without dystonia (see Table 2).

**Table 2:** Comparison of demographic, clinical and imaging characteristics between Progressive supranuclear palsy patients with and without dystonia.


VARIABLES	PATIENTS WITH DYSTONIA (n = 57)	PATIENTS WITHOUT DYSTONIA (n = 31)	P VALUE

Age of onset (Mean ± SD) (Years)	63.5 ± 7.2	70.1 ± 7.3	0.139

Gender (M/F)	63% M, 37% F	71% M, 29% F	0.982

Disease duration (Mean ± SD) (Years)	3.9 ± 2.3	3.6 ± 2.4	0.464

Distribution of PSP subtype	PSP-RS 51% (n = 29), PSP-CBS 31% (n = 18), PSP-P 16% (n = 9), PSP-PGF 2% (n = 1)	PSP-P 45% (n = 14), PSP-RS 19% (n = 6), PSP-F 13% (n = 4), PSP-CBS 10% (n = 3), PSP-C 6% (n = 2), PSP-PGF 3% (n = 1), PSP-SL 3% (n = 1)	**<0.001**

Mean MDS-UPDRS score	30.5 ± 5.7	30.3 ± 6.0	0.883

Mean MoCA score	17.8 ± 4.7	19.5 ± 3.7	0.08

M:P ratio	0.22 ± 0.08	0.27 ± 0.07	**0.005**

MRPI	17.7 ± 4.5	14.4 ± 2.6	**0.003**


**Footnotes**: PSP Progressive supranuclear palsy, PSP-RS Richardson’s syndrome, PSP-P Parkinsonism, PSP-F Frontal, PSP-PGF Progressive gait freezing, PSP-CBS Cortico-basal syndrome, PSP-C Cerebellar ataxia, PSP-SL Speech/language disorders, SD Standard deviation, M Male, F Female, MDS-UPDRS Movement Disorder Society-Unified Parkinson’s Disease Rating Scale, MoCA Montreal Cognitive Assessment, M:P Midbrain to pons ratio, MRPI Magnetic Resonance Parkinsonism Index.

However, subtype distribution differed significantly (p < 0.001), with dystonia more frequently observed in PSP-RS and PSP-CBS, whereas PSP-P and other subtypes were more common in patients without dystonia.

Among patients with available data on latency, the mean time to dystonia onset was 28.8 ± 21.2 months in PSP-RS, 30.0 ± 18.7 months in PSP-P, and 19.1 ± 19.2 months in PSP-CBS. Median latency was 24 months in PSP-RS and PSP-P, and 18 months in PSP-CBS. Although not statistically significant (p = 0.23), there was a trend toward earlier dystonia onset in PSP-CBS. Early dystonia (≤12 months) was observed in 42.9% of PSP-CBS patients compared to 20% in PSP-RS and PSP-P (p = 0.24). Dystonia was the presenting feature in five patients, four of whom had PSP-CBS.

Neuroimaging markers differed significantly, with lower M:P ratios (0.22 ± 0.08 vs 0.27 ± 0.07; p = 0.005) and higher MRPI values (17.7 ± 4.5 vs 14.4 ± 2.6; p = 0.003) in patients with dystonia, suggesting greater brainstem involvement.

### Phenomenology of Dystonia in PSP

Among patients with dystonia, 28 had isolated limb involvement, 14 had isolated cranio-cervical dystonia, and 15 had both limb and cranio-cervical involvement (see Table 3).

**Table 3:** Comparison of demographic, clinical and imaging characteristics across different dystonia patterns in Progressive supranuclear palsy.


VARIABLES	PATIENTS WITH LIMB DYSTONIA ONLY (n = 28)	PATIENTS WITH CRANIO-CERVICAL DYSTONIA ONLY (n = 14)	PATIENTS WITH BOTH LIMB AND CRANIO-CERVICAL DYSTONIA (n = 15)	P VALUE

Age of onset (Mean±SD) (Years)	62.6 ± 5.9	64.9 ± 9.5	66.6 ± 6.5	0.15

Gender (M/F)	71% M (n = 20), 29% F (n = 8)	57% M (n = 8), 43% F (n = 6)	53% M (n = 8), 47% F (n = 7)	0.3

Disease duration (Mean ± SD) (Years)	3.9 ± 2.0	3.1 ± 1.6	4.8 ± 2.9	0.09

Duration to dystonia onset (Mean ± SD) (Months)	22.1 ± 17.6	27 ± 14.5	28.2 ± 25.7	0.6

Distribution of PSP subtype	PSP-CBS 43% (n = 12), PSP-RS 36% (n = 10), PSP-P 18% (n = 5), PSP-PGF 3% (n = 1)	PSP-RS 86% (n = 12), PSP-P 14% (n = 2)	PSP-RS 47% (n = 7), PSP-CBS 40% (n = 6), PSP-P 13% (n = 2)	**0.02**

Mean MDS-UPDRS score	28.9 ± 5.7	31.5 ± 5.4	32.6 ± 4.7	0.81

Mean MoCA score	18.4.9 ± 4.8	17.2 ± 4.4	16.6 ± 4.7	0.73

M:P ratio	0.24 ± 0.07	0.19 ± 0.08	0.2 ± 0.09	0.11

MRPI	16.3 ± 3.1	19.4 ± 5.3	20.5 ± 6.4	0.22


**Footnotes**: PSP Progressive supranuclear palsy, PSP-RS Richardson’s syndrome, PSP-P Parkinsonism, PSP-PGF Progressive gait freezing, PSP-CBS Cortico-basal syndrome, SD Standard deviation, M Male, F Female, MDS-UPDRS Movement Disorder Society-Unified Parkinson’s Disease Rating Scale, MoCA Montreal Cognitive Assessment, L Limb, CC Cranio-cervical, M:P Midbrain to pons ratio, MRPI Magnetic Resonance Parkinsonism Index.

There were no significant differences in age at onset, gender distribution, or motor and cognitive scores across these groups. Disease duration showed a non-significant trend toward longer duration in patients with both limb and cranio-cervical dystonia (4.8 ± 2.9 years) compared to limb-only (3.9 ± 2.0 years) and cranio-cervical-only (3.1 ± 1.6 years) groups (p = 0.09).

Subtype distribution differed significantly (p = 0.021). Limb dystonia was most frequently associated with PSP-CBS (43%), followed by PSP-RS (36%). Cranio-cervical dystonia was predominantly seen in PSP-RS (86%), while both limb and cranio-cervical dystonia were distributed between PSP-RS (47%) and PSP-CBS (40%) (see Videos 3 and 4). Limb dystonia was present in 85.7% of PSP-CBS cases (n = 18/21), predominantly involving the upper limb at onset. Among the 29 patients with cranio-cervical dystonia, blepharospasm was present in 62% (n = 18/29), facial dystonia in 45% (n = 13/29), and orolingual dystonia in 31% (n = 9/29) patients. Some patients had more than one craniofacial dystonia subtype, most commonly a combination of blepharospasm and facial dystonia, while others also had concurrent orolingual involvement. Apraxia of lid opening was more frequent in PSP-RS (31.4%) compared to PSP-CBS (14.3%) and PSP-P (4.3%) (p = 0.03) (see Video 5).

**Video 3:** A patient with Progressive Supranuclear Palsy–Richardson’s Syndrome (PSP-RS), demonstrating predominant blepharospasm and bradykinesia on examination.

**Video 4:** A patient with Progressive Supranuclear Palsy–Richardson’s Syndrome (PSP-RS) demonstrating cervical and limb dystonia along with vertical supranuclear gaze palsy.

**Video 5:** A patient with Progressive Supranuclear Palsy–Richardson’s Syndrome (PSP-RS) demonstrating apraxia of lid opening, limb dystonia, and severe bradykinesia on examination.

Neuroimaging markers (M:P ratio and MRPI) did not differ significantly across dystonia patterns. As part of the therapeutic management of functionally disabling limb or cranio-cervical dystonia, botulinum toxin injections were administered in 19 of 57 patients with dystonia (33.3%). All injections were performed under ultrasound guidance. Clinical response was assessed at follow-up, based on combined physician assessment and patient-reported improvement. Twelve patients (63.2%) demonstrated marked improvement, while seven (36.8%) reported partial improvement in their symptoms of dystonia-related stiffness and pain. No formal outcome scales were used, and no patient reported worsening following botulinum toxin injection.

## Discussion

With the advent of newer diagnostic criteria, the clinical spectrum of PSP has expanded, allowing improved recognition of subtypes beyond the classical PSP-RS [6]. This classification is clinically relevant, as outcomes vary across PSP subtypes [20]. Subcortical variants such as PSP-P and PSP-PGF are associated with longer disease duration and slower progression compared to PSP-RS, which remains the most common and more aggressive phenotype [21]. While earlier literature has described PSP-CBS as relatively rare, nearly one-fourth of the cases in our prospective cohort were classified as PSP-CBS [9,22,23]. This higher frequency likely reflects systematic data collection and focused evaluation for dystonia and cortical features. As PSP-CBS is recognized as a distinct clinical phenotype within the 2017 MDS diagnostic criteria, its inclusion allowed evaluation of dystonia across the full spectrum of clinically recognized PSP variants. Although limb dystonia is a characteristic feature of PSP-CBS and may have contributed to the overall prevalence of dystonia, our subtype-specific analyses demonstrated distinct patterns of dystonia across the different PSP phenotypes. Within this evolving framework, dystonia emerges as an important yet under-recognized clinical feature of PSP.

We reviewed data from 29 studies evaluating dystonia in PSP. Most available literature consisted of case reports describing focal craniofacial manifestations, including facial, oromandibular, lingual, and laryngeal dystonia [24,25,26,27,28,29,30,31,32,33,34,35,36]. A few reports also described levodopa-induced or dopaminergic therapy-induced craniofacial and oromandibular dystonia [24,29,31,33]. Across published studies, the reported prevalence of dystonia in PSP ranged from approximately 30% to 60% (see Table 4) [14,15,16,17,37,38,39,40]. Much of the existing literature is retrospective and based on small cohorts, suggesting that dystonia may have been historically under-recognized and overshadowed by more prominent features such as vertical gaze palsy and postural instability [41]. Nevertheless, dystonia has clear clinical relevance, as it contributes to pain, impaired dexterity, and increased risk of falls, thus affecting the quality of life of the patient [42].

**Table 4:** Major published studies on dystonia in Progressive supranuclear palsy.


AUTHOR AND YEAR	NUMBER OF CASES	DYSTONIA PREVALENCE	PHENOMENOLOGY	ADDITIONAL FEATURES

Rafal 1987 [14]	N = 30 PSP cases	26.7% (n = 8)	Details not available	62.5% dystonia was not on any medication; 50% dystonia presented before ophthalmoplegia

Rinne 1994 [38]	N = 36 clinically probable/pathologically proven CBD	Fixed dystonic posture 18.5% (n = 6)	Details not available	Cortical sensory loss 25% (n = 9); unilateral ‘clumsy, stiff or jerky arm’ 55.6% (n = 20); gait difficulties and supranuclear ophthalmoplegia emerged after a mean follow-up of 5.2 years

Barclay 1997 [16]	N = 83 PSP cases	45.8% (n = 38)	24% blepharospasm, 27% limb dystonia, 17% axial dystonia in extension	Early dystonia 7.2% (n = 6); levodopa-induced dystonia 3.6% (n = 3), concurrent diseases 3.6% (n = 3) hydrocephalus, arteriosclerotic disease

Vanek 2001 [39]	N = 66 clinically probable/pathologically proven CBD	59% (N = 39)	Arm dystonia 92%; leg dystonia 28%; cranio-cervical and truncal dystonia 31%	51% had early dystonia in arm; 33% had initial leg involvement. Axial or leg dystonia, without significant arm involvement was rare.

Godeiro-Junior 2008 [17]	N = 38 (23 MSA; 7 CBD; 8 PSP)	Overall 50% MSA 30.4% (n = 7); PSP 62.5% (n = 5); CBD 100% (n = 8)	Camptocormia; retro/anterocollis; blepharospasm; oromandibular; limb dystonia	None had dystonia as first symptom; Levodopa therapy did not influence dystonia

Stamelou 2012 [37]	N = 296 pathologically proven CBD cases (54% CBS; 15% FTD; 10.7% PSP)	37.5% (n = 111)	CBS phenotype early upper limb dystonia; dementia phenotype late cranio-cervical dystonia	Akinetic-rigid syndrome 100%; apraxia 86.3%; myoclonus ~50%

Yoon 2018 [40]	N = 1278 patients with parkinsonism	Overall 13.8% (n = 176) PD 11%; MSA 20.9%; PSP 40.7%; CBD 66.7%	Cranio-cervical dystonia frequent in atypical parkinsonism; limb dystonia frequent in PD and CBD; truncal dystonia frequent in PD	Levodopa-related limb and truncal dystonia frequent in PD; levodopa non-related cranio-cervical dystonia frequent in atypical parkinsonism

Wagle-Shukla 2019 [15]	N = 217 PSP cases	30% (n = 66)	Cervical 50% (n = 33); blepharospasm, 25.8% (n = 17); limb 65.2% (n = 43); cranial (face, jaw, tongue) 6.1% (n = 4); generalized 7.6% (n = 5)	33% had tremor (n = 71); only 12% (n = 27) had both tremor and dystonia

Present study	N = 88 PSP cases	64.8% (N = 57)	Limb dystonia 49.1%; cranio-cervical 24.6%; both limb and cranio-cervical 26.3%	Limb dystonia frequent in PSP-CBS (43%); cranio-cervical dystonia frequent in PSP-RS (86%). Early dystonia common in PSP-CBS (42.9%); while PSP-P predominated in patients without dystonia. Patients with dystonia had lower M:P ratios and higher MRPI.


**Footnotes**: PSP Progressive supranuclear palsy, PSP-RS Richardson’s syndrome, PSP-P Parkinsonism, CBS Cortico-basal syndrome, CBD Cortico-basal degeneration, PD Parkinson’s disease, MSA Multiple system atrophy, M:P Midbrain to pons ratio, MRPI Magnetic Resonance Parkinsonism Index, n,N Number of cases.

Dystonia in PSP shows considerable heterogeneity in its distribution. Some studies report a predominance of cranio-cervical involvement, particularly blepharospasm and cervical dystonia, while others describe a higher frequency of limb dystonia [15,16,39]. In our cohort, limb-only dystonia was the most frequent pattern, followed by cranio-cervical dystonia and combined involvement. This variation likely reflects differences in cohort composition as well as the prospective nature of our study, which allowed systematic detection.

A clear relationship between dystonia patterns and PSP subtypes was observed in our study. Dystonia was more frequently associated with PSP-RS and PSP-CBS, whereas patients without dystonia were more commonly classified as PSP-P and other subtypes. Limb dystonia was strongly associated with PSP-CBS, while cranio-cervical dystonia was predominant in PSP-RS. Apraxia of lid opening was also more common in PSP-RS, in keeping with the existing literature describing blepharospasm and ALO as characteristic features of this subtype [43,44]. Importantly, dystonia appeared earlier in the disease course in PSP-CBS. Patients with PSP-CBS showed shorter latency to dystonia onset and a higher proportion of early dystonia. In several cases, dystonia was the presenting feature, most commonly in PSP-CBS. These findings suggest that focal limb dystonia may serve as an early clinical marker, even before overt cortical signs become apparent. Previous reports have similarly described asymmetric limb dystonia as an early manifestation in PSP-CBS and have documented cases in which dystonia preceded the development of ophthalmoplegia and gait impairment by several years [14,38]. However, few studies have systematically evaluated the latency to dystonia onset across PSP subtypes. Our findings therefore extend the existing literature by demonstrating subtype-specific differences in the temporal evolution of dystonia.

The prominence of limb dystonia in PSP-CBS also reflects the overlap between PSP and Corticobasal Degeneration. Features such as asymmetric limb dystonia, apraxia, and alien limb phenomenon are traditionally associated with CBS but are increasingly recognized in PSP-CBS [45,46,47]. Current diagnostic criteria do not explicitly incorporate asymmetric limb dystonia into the classification of possible PSP-CBS [6]. As a result, some patients presenting with early asymmetric dystonia without cortical signs may be misclassified as PSP-RS. This highlights the importance of longitudinal follow-up, as such cases may represent evolving PSP-CBS [48,49,50]. Moreover, Rafal and Friedman observed that the severity of limb dystonia did not correlate with the severity of ophthalmoplegia in their patients, suggesting that dystonia may evolve independently of the classical oculomotor involvement [14].

Consistent with previous literature, asymmetric upper limb dystonia was the most frequent manifestation in PSP-CBS, while lower limb and cranio-cervical involvement were less common [23,39,51]. Earlier reports have suggested that dystonia is less frequent than rigidity in PSP-CBS; however, this was not observed in our cohort, where the majority of PSP-CBS patients exhibited dystonia [52,53].

In contrast, PSP-P was less frequently associated with dystonia. Patients without dystonia were more commonly classified as PSP-P, which is consistent with previous literature describing this subtype as having fewer atypical features early in the disease course and often being mistaken for Parkinson’s disease (PD) [54,55]. The potential evolution of PSP-P into PSP-RS remains debated, and it is unclear whether this reflects true disease progression or initial diagnostic misclassification [56,57]. This distinction remains important for clinical interpretation and follow-up. Tremor, however, was most commonly observed in patients with PSP-P. In patients with PSP-CBS, distinguishing action tremor from cortical myoclonus may be challenging on clinical examination alone. As electrophysiological confirmation was not performed systematically, we cannot exclude the possibility that some cases clinically classified as action tremor represented cortical myoclonus. Future studies incorporating electrophysiological assessment are warranted to better characterize these hyperkinetic manifestations.

The pattern of dystonia also appeared to relate to the disease duration. Patients with combined limb and cranio-cervical involvement had longer disease duration compared to those with focal dystonia, suggesting that dystonia may evolve and generalize over time, reflecting wider neuroanatomical involvement as the disease progresses. However, disease duration did not differ significantly between patients with and without dystonia overall. Therefore, although progressive network involvement may contribute to changes in the distribution of dystonia, the cross-sectional design precludes conclusions regarding whether longer disease duration directly influences the development or progression of dystonia.

Neuroimaging findings supported these clinical observations. Patients with dystonia demonstrated greater brainstem involvement, as indicated by lower midbrain-to-pons ratios and higher MRPI values. This suggests more severe degeneration in brainstem structures.Although these imaging markers are well established for supporting the diagnosis of PSP, their relationship with dystonia has not been systematically evaluated. One possible explanation is that more extensive degeneration involving the midbrain, basal ganglia, and their interconnected brainstem networks disrupts motor control circuits, thereby predisposing to the development of dystonia. In PSP-CBS, cortical atrophy was also observed in the contralateral parietal region, corresponding to the side of limb dystonia. These findings support the role of distributed network involvement, including basal ganglia, brainstem, and cortical regions, in the pathogenesis of dystonia.The relationship between dystonia and dopaminergic therapy in PSP remains uncertain. Earlier reports have suggested that dystonia may be induced or exacerbated by levodopa in some cases, but this association has not been consistent with several case series describing this as a rare occurrence [14,16,17,33]. In our cohort, no clear relationship was observed between dystonia and levodopa exposure, with several patients being levodopa-naïve at the time of onset. This supports the view that dystonia in PSP is more likely a manifestation of the underlying disease rather than a treatment-related effect, in contrast to PD [40].

The coexistence of parkinsonism and dystonia represents a clinical paradox. Parkinsonism is hypokinetic, whereas dystonia is hyperkinetic. This combination likely reflects dysfunction across interconnected basal ganglia, brainstem, and cortical circuits, rather than a simple dopaminergic deficit [58,59]. Such network-level involvement explains why dystonia is frequently observed across atypical parkinsonian syndromes, including PSP [60,61,62].

Therapeutic management in PSP remains challenging due to widespread pathological involvement and clinical complexity [63]. In this context, targeted symptomatic therapies are important [64,65,66]. Botulinum toxin was used in one-third of our patients and resulted in meaningful clinical improvement. These findings support its role as an effective symptomatic treatment for dystonia, helping reduce pain and stiffness and improving the quality of life in selected patients [67].

Studies from South Asian cohorts have highlighted considerable phenotypic variability in PSP, with a broader spectrum of clinical features than traditionally described [68]. Our findings add to this observation, demonstrating a relatively higher proportion of PSP-CBS and a strong association between dystonia patterns and specific subtypes. This underscores the importance of careful phenotypic characterization and prospective evaluation. Taken together, these findings support the concept of a “dystonia spectrum” in PSP, encompassing cranio-cervical and limb manifestations, and reflecting underlying neuroanatomical involvement.

This study has certain limitations. The absence of pathological confirmation limits diagnostic certainty [69]. In addition, patients were assessed at a single time point, which precludes evaluation of the longitudinal evolution of dystonia and potential transitions between PSP subtypes. Furthermore, dystonia severity and its functional impact were not systematically assessed using validated dystonia-specific rating scales, limiting our ability to quantify its effect on disability and daily functioning. Future longitudinal and clinicopathological studies are needed to better understand the natural history of dystonia in PSP and its relationship with disease progression and underlying tau pathology.

## Conclusion

Dystonia is a common and clinically significant feature in PSP, with distinct phenotypic patterns across subtypes. The differential distribution of limb and cranio-cervical dystonia, along with a trend toward earlier onset in PSP-CBS, highlights its potential value as a clinical marker. Importantly, targeted interventions such as botulinum toxin can provide meaningful symptomatic relief. Overall, our findings support a “dystonia spectrum” in PSP with distinct clinical and anatomical correlates. Future longitudinal and clinicopathological studies are warranted to better delineate its evolution and underlying disease mechanisms.
